# 

**Published:** 1898-12-24

**Authors:** 


					1THE HOSPITAL. " The standard of highest purity."?Lancet, Deo
CADBURY'S O IfifH CADBURY'S
coooa m, Mm JeK ooooa
ABSOLUTELY PURE. NO DRUGS OR ALKALIES USED.
iW$!
mi
IV*v /?? ??riavtuu IDLFLRMAJUf
^639. Vol, XXV. [S?] Saturday
Dec. 24th, 1898.
-? ~=HOAf.OLKAMB-MO/i W1CH HOSP.LTAJ, .

				

